# Biologic Agents in Inflammatory Eye Disease

**Published:** 2011-10

**Authors:** Chiara Posarelli, Ilir Arapi, Michele Figus, Piergiorgio Neri

**Affiliations:** 1 Department of Neurosciences, Ophthalmology, University of Pisa, Pisa, Italy; 2 The Eye Clinic, Polytechnic University of Marche, Ancona, Italy; 3 Ophthalmology/ Ear, Nose, and Throat Department, Mother Teresa University Hospital Center, Tirana, Albania

**Keywords:** Biologic Agent, Immunosuppression, Uveitis

## Abstract

Non-infectious uveitis is a potentially sight threatening disease. Along the years, several therapeutic strategies have been proposed as a means to its treatment, including local and systemic steroids, immunosuppressives and more recently, biologic agents. The introduction of biologics can be defined as a new era: biologic therapies provide new options for patients with refractory and sight threatening inflammatory disorders. The availability of such novel treatment modalities has markedly improved the therapy of uveitis and considerably increased the possibility of long-term remissions. This article provides a review of current literature on biologic agents, such as tumor necrosis factor blockers, anti-interleukins and other related biologics, such as interferon alpha, for the treatment of uveitis. Several reports describe the efficacy of biologics in controlling a large number of refractory uveitides, suggesting a central role in managing ocular inflammatory diseases. However, there is still lack of randomized controlled trials to validate most of their applications. Biologics are promising drugs for the treatment of uveitis, showing a favorable safety and efficacy profile. On the other hand, lack of evidence from randomized controlled studies limits our understanding as to when commence treatment, which agent to choose, and how long to continue therapy. In addition, high cost and the potential for serious and unpredictable complications have very often limited their use in uveitis refractory to traditional immunosuppressive therapy.

## INTRODUCTION

Intraocular inflammation is one of the leading causes of visual impairment and can be divided into non-infectious and infectious uveitis. Treatment for the former category is characterized by two phases of acute stage and maintenance therapy. The acute stage can be successfully controlled with the use of pharmacologic agents such as corticosteroids but when this phase is controlled, reduction in steroid dose is mandatory. Long-term treatment with steroids entails a certain number of systemic and ocular side effects such as hypertension, diabetes, cataracts and glaucoma. In cases of steroid dependency and high disease activity, immunotherapy is often initiated, however the treating physician frequently balances side effects with the therapeutic response. An increased understanding of mechanisms that result in non-infectious uveitis has made it possible to consider other means to abrogate the ocular immune response. After the advent of molecular biology techniques in the early 90s, several immunosuppressive agents have been proposed for treatment of ocular inflammation. Biologics are highly specific molecules targeting soluble inflammatory mediators; these include recombinant antibodies to, or antagonists of, particular cytokines or cell-surface receptors, and recombinant cytokines (such as interferons).

Biologic agents are promising drugs targeting specific molecules involved in inflammatory processes (Table 1). Over the last few years there have been a large number of reports on the use of biologic therapies to treat uveitis.^1^ Studies on anti-tumor necrosis factor (TNF)-α therapies and recombinant interferon (IFN)-α dominate the uveitis literature, with a limited number of reports on monoclonal antibody therapies against interleukins (for example IL-2 and IL-1b) and the vascular endothelial growth factor (VEGF). Results regarding their efficacy in uveitis are very promising, but each agent shows potential adverse effects and furthermore, the uveitis literature is mostly composed of uncontrolled trials or case-series. Many questions still remain unanswered about biologics: when to begin therapy, which agent and dose to use, and when to discontinue treatment. Due to high cost and limited long-term experience with biologic therapies, they are reserved for uveitis refractory to traditional immunosuppressives. However, the efficacy of these agents may justify their use earlier in the therapeutic process, as in Behcet’s disease.^2^

In the present review we will summarize the most relevant applications of biologics in uveitis.

## ANTI-TUMOR NECROSIS FACTOR ALPHA THERAPIES

TNF-α is a key cytokine involved in the pathogenesis of many inflammatory disorders, including non-infectious uveitis. TNF-α is generated and expressed by immune cells and binds to the corresponding TNF receptor (TNFR) family. This cytokine has affinity for two receptors, known as p55 or TNF-R1, and p75 or TNF-R2^3^,^4^ leading to signal transduction and stimulation of inflammation in autoimmune reactions. TNF-α activates T-cells and macrophages, by increasing the expression of endothelial adhesion molecules and pro-inflammatory cytokines.^3^,^5^,^6^

TNF-α plays a key role in the pathogenesis of many inflammatory diseases; it has been detected in all tissues affected by active inflammation, such as the synovial fluid in patients with rheumatoid arthritis (RA) or psoriasis arthritis (PsA), the bowel mucosa in Crohn’s disease or ulcerative colitis, and the eye during acute uveitis.

Non-infectious intermediate, posterior, and panuveitis^7^,^8^ are antigen-specific CD4 T-cell–mediated autoimmune diseases. In these conditions, TNF-α represents one of the most important amplifying factors in inflammatory reactions^9^–^11^; in case of uveitis, TNF-α is found at high concentrations both in the aqueous humor and in the serum^12^–^16^, similar to RA^17^.

Currently there are three anti-TNF-α agents available: etanercept (a recombinant fusion protein, combining two human p75 TNF-α receptors linked to the Fc domain of human IgG1)^18^, infliximab (a mouse–human chimeric monoclonal IgG1 antibody against TNF-α)^19^, and adalimumab (a fully humanized monoclonal IgG1 antibody against TNF-α). Infliximab and adalimumab bind effectively both to soluble and trans-membrane TNF-α. In contrast etanercept forms less stable bonds with the transmembrane form of TNF-α.^20^

Unfortunately, data emerging from the literature on etanercept are contradictory. Although etanercept has been proven to be effective in uveitis associated with different systemic disorders^21^–^24^, both infliximab and adalimumab have shown better control of uveitis compared to etanercept in retrospective studies and questionnaire-based surveys^25^–^29^. In addition, etanercept seems to be associated with exacerbation of uveitis and “de novo” induction of uveitis.^30^

Infliximab, the first commercially available anti-TNF-α agent, has been shown to be effective treatment for both anterior and posterior uveitis^2^,^25^,^27^–^29^,^31^–^39^. Infliximab is administered intravenously, at a usual dose of 3–10 mg/ kg, but doses as high as 10–20 mg/kg have been reported with success and few side effects in a group of 17 children with chronic uveitis.^36^ Behcet’s disease related posterior uveitis, seems to respond rapidly to infliximab infusions (range: less than 7 days to 3 weeks), as confirmed by the open-label cohort study by Tugal-Tutkun et al^34^, while non-Behcet’s posterior uveitis responds more variably (range: 6 days to 2 months)^2^.

Summarizing recent reports on infliximab therapy in refractory posterior uveitis suggests that in 78–100% of cases, an initial response to therapy was achieved^2^,^25^,^27^,^31^–^37^; however, the effect is temporary and repeated infusions are necessary every 4–8 weeks to maintain remission.

The high percentage of response to infliximab is encouraging, as all patients in these studies previously failed to respond to traditional therapies. Despite increasing the dose and frequency of infusions, some patients suffer from relapses or infusion related allergic reactions; this may be explained by the development of antibodies against the murine component of the molecule. For this reason, many clinicians prescribe concomitant therapy with an antimetabolite or glucocorticoid. Moreover, it is still not clear whether infliximab should be infused at variable or regular intervals^40^,^41^, in order to reduce allergic reactions and optimize the long term outcome.

There are several serious adverse events related to infliximab therapy, including an increased risk of malignancies, tuberculosis, multiple sclerosis, and lupus-like reactions, therefore such therapy should be avoided in patients with a positive history of these conditions.

Suhler et al^33^ reported a greater incidence of adverse events than any other recent study with six out of 23 (26%) patients developing possible infliximab related adverse events requiring withdrawal of treatment. This study also examined visual fields and electroretinograms with no significant deterioration at 1-year of follow up.

Infliximab showed efficacy for treatment of refractory uveitis associated with juvenile onset rheumatological disease, even in cases where previous biologics had failed.^42^

Adalimumab, a recombinant IgG1 monoclonal antibody targeting TNF-α, showed efficacy both as monotherapy and in combination with other disease-modifying anti-rheumatic drugs (DMARDs), with favorable safety and efficacy profile in inflammatory rheumoarthropaties of different etiologies. Adalimumab has been proven to be effective in adults for treatment of RA^43^, ankylosing spondylitis (AS)^44^ and PsA^45^ by reducing symptoms of joint involvement and by inhibiting the progression of structural damage, typical of these immune-mediated diseases. Unlike infliximab and other biologic agents which have to be administered intravenously, adalimumab has the advantage of subcutaneous administration resulting in smoother drug levels over time, and convenient self-administration at home. The usual dose is 40mg at 2-week intervals. Adalimumab is produced by recombinant deoxyribonucleic acid (DNA) technology, called “phage display”, and is classified as a fully humanized antibody, even though its humanization is not total. Mushtaq et al^46^ described three patients with quiescent panuveitis associated with Behcet’s disease, who were successfully switched from infliximab to adalimumab. All three patients remained in remission after 11–24 months of adalimumab therapy and there were no serious adverse events. In childhood uveitis,^47^,^48^ adalimumab shows 80–88% reduction in inflammation, with many patients being able to reduce or stop concomitant corticosteroids and second line immunosuppressives. In these reports, with a total of 33 patients, there were no adverse reactions other than pain and burning at the injection sites. Unfortunately the anatomical site of uveitis was not described, but this may be presumed to be mainly anterior as the majority of children had juvenile idiopathic arthritis (JIA). The results of these studies suggest that adalimumab may be a reasonable alternative to infliximab in the treatment of uveitis, but no studies have yet compared these agents.^49^ Until now, adalimumab has mostly been given in cases of failure to respond to other anti-TNF-α agents, or because of convenient administration.

## ANTI-INTERLEUKIN THERAPIES

In the pathophysiology of posterior uveitis, some experimental models of autoimmune uveitis have implicated the role of interleukin (IL)-1 and IL-2. Daclizumab is a humanized monoclonal antibody against the IL-2 receptor (particularly the 55 kDa chain of the IL-2 receptor complex known as the Tac or CD25 subunit). Nussenblatt et al^50^ described the use of 4-weekly intravenous infusions of daclizumab for treatment of 10 patients with severe sight threatening intermediate and posterior uveitis. Eight out of 10 patients showed good control of disease over a 12-month period with concomitant tapering of standard immunosuppressive therapy. Similar results were observed by Hernandez Garfella et al^51^ who described reduced inflammatory activity in 70% of patients treated with intravenous daclizumab for 2 years, affected by refractory uveitis. Recently an open label study reported that daclizumab was able to stabilize visual acuity in 67% of patients with non-infectious intermediate, posterior, or panuveitis, concomitant with reduction in other immunosuppressives^52^. In this series, no serious adverse events were reported.

IL-1 receptor antagonist (IL-1RA) is a naturally occurring inhibitor of IL-1. Anakinra is a recombinant human IL-1RA, administered at a daily dose of 100 mg subcutaneously in adults. A case report described successful treatment with anakinra for chronic infantile neurological cutaneous articular syndrome (CINCA)-associated uveitis, refractory to anti-TNF therapy. This confirms the success observed in a preclinical experimental autoimmune uveitis model in mice.^54^ For such reasons, anakinra may be considered as an alternative biologic agent in patients with JIA-associated uveitis refractory to anti-TNF therapy.

## INTERFERONS

Although not properly a biologic agent, particular attention should be paid to a very interesting and promising drug, which is playing an important role in the management of severe sight threatening uveitis: interferon alpha (IFN-α). IFN-α is a naturally occurring cytokine secreted in response to viral infections, primarily by plasmacytoid dendritic cells. In simple terms, IFN-α is proposed as the primary pathogenic cytokine in ‘systemic’ autoimmune diseases, whereas TNF-α is believed to be the more pathogenic cytokine in organ-specific autoimmune diseases. TNF-α and IFN-α cross regulate each other; TNF-α reduces IFN-α levels by inhibiting the number and function of plasmacytoid dendritic cells.^55^

Recent studies have revealed many similarities between TNF-α blockers and IFN-α therapies for uveitis. Both agents have a rapid effect on intraocular inflammation and achieve control of uveitis in a high percentage of patients that have failed to respond to traditional second-line immunosuppressives.

Recombinant human IFN-α2a and IFN-α2b have both been used to treat posterior uveitis successfully, with the majority of studies using IFN-α2a. IFN-α^56^ is given by subcutaneous injections, commonly starting with high dose daily injections (6 mU per day) with a subsequent taper to low dose intermittent injections (3 mU, two or three times per week). It is a standard procedure to discontinue second-line immunosuppressives prior to IFN-α therapy, and many ophthalmologists also taper corticosteroids to the lowest dose possible.

The major experience with the use of IFN-α therapy involves patients affected with Behçet’s disease with response rates of 83 to 92%, within 2 to 4 weeks.^57^–^59^ Recently, several reports have also included patients with other causes of uveitis including, sympathetic ophthalmia, Vogt-Koyanagi-Harada disease, birdshot chorioretinopathy, intermediate uveitis, and idiopathic panuveitis^57^,^61^.

IFN-α therapy can usually be stopped after 6–12 months of relapse-free status: 20–40% of patients remained quiescent 7 to 58 months after IFN-α treatment^58^,^59^.

IFN-α precipitates a flu-like syndrome during the first week of treatment in almost 100% of patients, and depression has been reported in 4–7.5% of subjects.^58^,^59^,^61^ Neutropenia, alopecia, elevated liver enzymes, epilepsy and injection site ulcers are potentially severe sequelae, causing discontinuation of treatment. IFN-β has also been reported as a useful treatment for intermediate uveitis associated with multiple sclerosis, choroiditis and choroidal neovascularization in chronic recurrent inner choroidopathy.^62^,^63^

## OTHER BIOLOGIC AGENTS

There are other monoclonal antibodies that have been used in uveitis refractory to standard immunosuppressive therapies; alemtuzumab, also known as Campath-1H, is a humanized monoclonal antibody against the pan-lymphocyte antigen CD52. It has been used for five consecutive days by intravenous infusion and seems to be an effective treatment, leading to long term remission as shown in a report published in 2000.^64^ However, Campath-1H causes significant lymphopenia for several weeks exposing the patient to a higher risk of infections.

Rituximab is a mouse–human chimeric monoclonal IgG1 antibody against CD20, expressed by B-cells. It has been reported as an effective treatment for scleritis associated with Sjogren’s syndrome^65^ and Wegener’s granulomatosis^66^. Evidence that rituximab is effective in conditions traditionally considered as predominantly T cell mediated autoimmune diseases, such as rheumatoid arthritis and Wegener’s granulomatosis, suggests that this drug may also play a role in treating refractory uveitis. Rituximab may represent rescue therapy for severe JIA-associated uveitis refractory to traditional immunosuppressives and TNF-α inhibitors.^67^

## CONCLUSION

There has been a great deal of hope since the medical literature witnessed reports on successful results with several biologic agents for treatment of refractory inflammatory disorders such as RA, JIA, seronegative arthropathies and inflammatory bowel disease. This enthusiasm has extended to ophthalmology as remarkable remissions have been observed in patients with ocular inflammation failing all other treatments, particularly in patients with Behçet’s disease. Patients with refractory uveitis, like those with systemic autoimmune diseases, may have much to gain from treatment with biologic agents. However, despite the theoretical rationale, lack of evidence from randomized controlled studies limits our understanding of when to commence therapy, which agent to choose and how long to continue treatment. In addition, high cost and potential side effects of biologic drugs have limited their use in uveitis refractory to traditional immunosuppression. Fortunately many biologic agents are licensed for several systemic conditions associated with uveitis, such as AS and JIA, and access to biologic therapy in such patients can be less problematic.

Research networks should be established to conduct randomized controlled trials comparing the efficacy of various biologic therapies. The results of such trials would provide evidence-based data for clinical decisions, regarding the optimal choice of therapy for long-term disease remission.

## Figures and Tables

**Table 1:** List of the most relevant biologic agents used in ophthalmology.

Name/Commercial name	Indications	Technology	Mechanism of action
Adalimumab/Humira	rheumatoid arthritis, ankylosing spondylitis, psoriatic arthritis, psoriasis, Crohn’s disease	monoclonal antibody	TNF antagonist
Infliximab/Remicade	rheumatoid arthritis, ankylosing spondylitis, psoriatic arthritis, psoriasis, Crohn’s disease	monoclonal antibody	TNF antagonist
Anakinra/Kineret	rheumatoid arthritis	recombinant human interleukin-1 receptor antagonist	Interleukin-1 receptor binder
Daclizumab/Zenapax	prevention of renal transplant rejection	monoclonal antibody	Interleukin-2 receptor binder
Abatacept/Orencia	rheumatoid arthritis	immunoglobulin CTLA-4 fusion protein	T-cell deactivation
Rituximab/MabThera	CD20-positive non-Hodgkins lymphoma, chronic lymphocytic leukemia, rheumatoid arthritis	monoclonal antibody	CD20 antigen binder
Alemtuzumab/Campath-1H	B-cell chronic lymphocytic leukemia (B-CLL)	monoclonal antibody	CD52 antigen binder
